# Gold Nanoparticle-Quantum Dot Fluorescent Nanohybrid: Application for Localized Surface Plasmon Resonance-induced Molecular Beacon Ultrasensitive DNA Detection

**DOI:** 10.1186/s11671-016-1748-3

**Published:** 2016-11-25

**Authors:** Oluwasesan Adegoke, Enoch Y. Park

**Affiliations:** 1 Laboratory of Biotechnology, Research Institute of Green Science and Technology, Shizuoka University, 836 Ohya, Suruga-ku, Shizuoka 422-8529 Japan; 2 Laboratory of Biotechnology, Department of Bioscience, Graduate School of Science and Technology, Shizuoka University, 836 Ohya, Suruga-ku, Shizuoka 422-8529 Japan

**Keywords:** Gold nanoparticle, Zeta potential, Biosensor, Colloidal stability, Quantum dot, DNA detection

## Abstract

In biosensor design, localized surface plasmon resonance (LSPR)-induced signal from gold nanoparticle (AuNP)-conjugated reporter can produce highly sensitive nanohybrid systems. In order to retain the physicochemical properties of AuNPs upon conjugation, high colloidal stability in aqueous solution is needed. In this work, the colloidal stability with respect to the zeta potential (ZP) of four negatively charged thiol-functionalized AuNPs, thioglycolic (TGA)-AuNPs, 3-mercaptopropionic acid (MPA)-AuNPs, l-cysteine-AuNPs and l-glutathione (GSH)-AuNPs, and a cationic cyteamine-capped AuNPs was studied at various pHs, ionic strength, and NP concentration. A strong dependence of the ZP charge on the nanoparticle (NP) concentration was observed. High colloidal stability was exhibited between pH 3 and 9 for the negatively charged AuNPs and between pH 3 and 7 for the cationic AuNPs. With respect to the ionic strength, high colloidal stability was exhibited at ≤10^4^ μM for TGA-AuNPs, l-cysteine-AuNPs, and GSH-AuNPs, whereas ≤10^3^ μM is recommended for MPA-AuNPs. For the cationic AuNPs, very low ionic strength of ≤10 μM is recommended due to deprotonation at higher concentration. GSH-AuNPs were thereafter bonded to SiO_2_-functionalized alloyed CdZnSeS/ZnSe_1.0_S_1.3_ quantum dots (SiO_2_-Qdots) to form a plasmon-enhanced AuNP-SiO_2_-Qdots fluorescent nanohybrid. The AuNP-SiO_2_-Qdots conjugate was afterward conjugated to a molecular beacon (MB), thus forming an ultrasensitive LSPR-induced SiO_2_-Qdots-MB biosensor probe that detected a perfect nucleotide DNA sequence at a concentration as low as 10 fg/mL. The limit of detection was ~11 fg/mL (1.4 fM) while the biosensor probe efficiently distinguished between single-base mismatch and noncomplementary sequence target.

**Electronic supplementary material:**

The online version of this article (doi:10.1186/s11671-016-1748-3) contains supplementary material, which is available to authorized users.

## Background

The use of nanoparticles (NPs) in the field of chemical and biological-related applications has been unprecedented [1–3]. In particular, thiol-functionalized gold nanoparticles (AuNPs) have been highly utilized in medical therapies, diagnostics, and biological imaging [4–6]. The ease of synthesis, the unique physico-chemical properties (with respect to surface plasmonic feature), biocompatibility, and surface functionalization feature have made AuNPs widely applicable in sensor/biosensor design [6]. Due to their inherent multivalent surface nature, therapeutic and diagnostic applications of functionalized AuNPs have been extensively explored [7, 8]. Passivation with small thiol ligands is one of the most efficient strategies to stabilize the surface of AuNPs as it minimizes nonspecific binding and helps to aid biocompatibility.

Apart from the stabilizing effects of thiol ligands, the terminal functional group moieties allow for conjugation/bioconjugation of AuNPs with other molecules of interest in order to form hybrid systems. Thiol AuNPs-based hybrids exhibit the optical properties of both the NP and the bonded molecule which ultimately has a stimulating effect on the targeted application. The use of thiol AuNP-based hybrids cuts across different spheres of chemical and biological domains. For example, tamoxifen-poly(ethylene glycol)-conjugated thiol AuNP was used to selectively target estrogen receptor of positive breast cancer cells [9]. Wei et al. have reported the utilization of thiol-poly(ethylene glycol)-functionalized Au nanorods for selective photothermal and selective uptake of cancer cell [10, 11] and in the field of sensor/biosensor design, antibody-conjugated thiol-AuNPs have been used for virus detection [12], while conjugation to multiwall carbon nanotubes have been used for human serum albumin detection [13].

High colloidal stability of thiol-capped AuNPs prior to conjugation is needed to ensure that the physicochemical properties of the NP are not lost upon binding with external entities. Since chemical and biological systems are known to respond to the stability of NPs with different physicochemical responses [14], it is therefore crucial to explore the colloidal stability of AuNPs prior to conjugation, which may aid appropriate selection of the NP for the targeted application. Obtaining accurate data with reliable output efficiency when utilizing AuNPs within biological systems depends on appropriate characterization processes. Adequate characterization helps not only to unravel the reactivity of the NPs but also to understand its physico-chemical function. Zeta potential (ZP) is a powerful technique used in determining surface charge of NPs. Within the pharmaceutical domain, ZP has emerged as a useful tool in unraveling the physico-chemical effects of NPs [15–18]. In this study, prior to conjugation of the thiol-capped AuNPs for biosensor application, we have comparatively probed the ZP of negatively charged thioglycolic acid (TGA)-, 3-mercaptopropionic acid (MPA)-, l-cysteine- and l-glutathione (GSH)-capped AuNPs, and cationic cysteamine-capped AuNPs.

In this work, the GSH-capped AuNP was selected based on its higher colloidal stability and conjugated to compact silica (SiO_2_)-functionalized CdZnSeS/ZnSe_1.0_S_1.3_ alloyed quantum dots (Qdots) to form a novel fluorescent AuNP-SiO_2_ CdZnSeS/ZnSe_1.0_S_1.3_ Qdots nanohybrid system. The AuNP-SiO_2_-CdZnSeS/ZnSe_1.0_S_1.3_ Qdots nanohybrid was utilized as a fluorescent signal generator in a molecular beacon (MB) biosensor assay for ultrasensitive DNA detection. The developed biosensor operates based on hybridization of the target DNA with the loop sequence of the MB. Upon hybridization, localized surface plasmon resonance (LSPR) signal from bonded AuNP triggers fluorescence enhancement changes in the Qdots in proportion to the concentration of the targeted DNA. DNA was chosen as a model nucleic acid analyte to test the efficacy of the biosensor. Our LSPR-induced Qdots-MB biosensor can detect ultrasensitive concentration of a perfect complementary DNA and distinguishes between single nucleotide mismatch and noncomplementary sequence. In general, our work is the first to explore a nanohybrid AuNP-SiO_2_ CdZnSeS/ZnSe_1.0_S_1.3_ Qdot fluorophore signal system in a MB assay for DNA detection.

## Methods

### Materials

Chlorotrimethylsilane, tetramethylammonium hydroxide pentahydrate (TMAH), cadmium oxide (CdO), octadecene (ODE), zinc oxide (ZnO), trioctylphosphine oxide (TOPO), selenium (Se), (3-aminopropyl)trimethoxysilane (3-APTMS), trioctylphosphine (TOP), hexadecylamine (HDA), sulfur, succinic anhydride, rhodamine 6G, *N*-(3-dimethylaminopropyl)-*N*′-ethylcarbodiimide hydrochloride (EDC), *N*-hydroxysuccinimide (NHS), HAuCl_4_·3H_2_O, tannic acid, TGA, MPA, l-cysteine, and GSH were purchased from Sigma Aldrich Co. LLC. (Saint Louis, MO, USA). Oleic acid was purchased from Nacalai Tesque Inc. (Kyoto, Japan). Methanol, tri-sodium citrate and potassium hydroxide (KOH), methanol, acetone, and chloroform were purchased from Wako Pure Chemical Ind. Ltd. (Osaka, Japan). An ultrapure Milli-Q Water System was used for sample preparation. The MB and synthetic DNA targets were purchased from FASMAC Co. Ltd. (Atsugi, Kanagawa, Japan). Black hole quencher-2 (BHQ-2) was used as the fluorescence quencher.

MB sequence: 5′-/NH_2_/**GCGAC**TTTCAGTTATTATGCCGTTGTATTT**GTCGC**/BHQ-2/-3′

Full complementary DNA: AAATACAACGGCATAATAACTGAAA

Single nucleotide DNA: AAATACAACG**T**CATAATAACTGAAA

Noncomplementary DNA: TGAAGCTAACCGGTAAGCGCTATAG

The bold sequence is the stem sequence of the MB.

### Characterization

UV/vis absorption and fluorescence emission measurements were performed using a filter-based multimode microplate reader (Infinite® F500, TECAN, Ltd., Männedorf, Switzerland). Powder X-ray diffraction (PXRD) measurements were carried out using a RINT ULTIMA XRD (Rigaku Co., Tokyo, Japan) with a Ni filter and a Cu-Kα source. Data were collected from 2 theta = 5–60° at a scan rate of 0.01°/step and 10 s/point. Transmission electron microscopy (TEM) images were obtained using a TEM JEM-2100F, (JEOL, Ltd., Tokyo, Japan) operated at 100 kV. The instruments used in this work for ZP and dynamic light scattering (DLS) analyses were conducted using a Zetasizer Nano series (Malvern Inst. Ltd., Malvern, UK). Data analysis was performed using the Malvern Instrument Dispersion Technology software (version 7.1).

### Synthesis of Thiol-capped AuNPs

Citrate-capped AuNPs were first synthesized according to literature procedure [19]. Then a ligand exchange reaction, replacing the citrate capping with TGA, MPA, l-cysteine, and GSH, was carried out by our own method [20]. A thiol-KOH-methanolic solution was first prepared. For the ligand exchange reaction with MPA, 3 g of KOH was dissolved in 40 mL of methanol and afterward 2 mL of MPA was added and the solution was stirred at an ambient temperature. Whereas, for the ligand exchange reaction with TGA, l-cysteine, and GSH, 1.5 g of KOH was dissolved separately in 20 mL of methanol and 1 mL of TGA and 1 g of l-cysteine and GSH were each added to separate KOH-methanolic solution. The citrate-capped AuNP solution was added into each of the thiol-KOH-methanol solution and stirred for few minutes at ambient temperature. The thiol-functionalized AuNPs were purified by centrifugation at 1500*g* for 10 min. The thiol-capped AuNP filtrate was further dissolved in ultrapure deionized water.

Cationic cysteamine-capped AuNPs were synthesized by mixing 1 mL of 1% HAuCl_4_·3H_2_O with 40 mL of water. After stirring for ~5 min, 500 μl of 1% cysteamine and 400 μl of 1% NaBH_4_ were added. Formation of cationic cysteamine-capped AuNPs was evident by the steady change in color of the solution from yellow to deep purple with time. The cysteamine-AuNPs were purified using acetone and acetone/chloroform mixture and stored at 4 °C prior to use.

The concentration of the stock AuNPs were determined from their absorption spectra according to literature procedure [21]. The concentration obtained are 77 nM for TGA-AuNPs, 34 nM for MPA-AuNPs, 25 nM for l-cysteine-AuNPs, 29 nM for GSH-AuNPs, and 535 nM for cysteamine-AuNPs.

### Preparation of SiO_2_-capped CdZnSeS/ZnSe_1.0_S_1.3_ Qdots

SiO_2_-capped CdZnSeS/ZnSe_1.0_S_1.3_ Qdots were prepared by silanization [22, 23] of TGA-capped alloyed CdZnSeS/ZnSe_1.0_S_1.3_ Qdots. The synthesis of alloyed TGA-capped CdZnSeS/ZnSe_1.0_S_1.3_ Qdots has been reported by our group [20]. Briefly, amino-SiO_2_-CdZnSeS/ZnSe_1.0_S_1.3_ Qdots was prepared by the reaction of 10 mL aqueous solution of TGA-capped CdZnSeS/ZnSe_1.0_S_1.3_ Qdots with 3-APTMS and methanol at ambient temperature. Quenching of the reaction was performed by adding methanol and chlorotrimethylsilane basified with TMAH pentahydrate. The amino-SiO_2_-CdZnSeS/ZnSe_1.0_S_1.3_ Qdots were purified using acetone and chloroform. Carboxyl-silanized CdZnSeS/ZnSe_1.0_S_1.3_ Qdots were prepared by suspension of the wet precipitate of amino-SiO_2_-CdZnSeS/ZnSe_1.0_S_1.3_ Qdots in buffer solution at pH 9 and succinic anhydride was added to generate the carboxyl group and stirred overnight. The carboxyl-silanized CdZnSeS/ZnSe_1.0_S_1.3_ Qdots were purified using acetone and chloroform. SiO_2_-Qdots are used to denote the carboxyl-silanized CdZnSeS/ZnSe_1.0_S_1.3_ Qdots.

### Conjugation Procedure

Conjugation of the SiO_2_-Qdots to GSH-AuNPs was performed by mixing 1 mL aqueous solution of 0.1 M EDC to 2 mL aqueous solution of the SiO_2_-Qdots (4 mg/mL) to activate the carboxylate group. The mixture was stirred for ~1 h after which 2 mL solution of GSH-AuNPs (4 nM) was added followed swiftly by the addition of 1 mL 0.1 M NHS. The SiO_2_-Qdots-AuNP nanohybrid conjugate was purified by centrifugation at 1500*g* for 3 min via a Nanosep® centrifugal filter having a 30,000 micron molecular weight cut-off (Pall Co., Port Washington, NY, USA).

The SiO_2_-Qdots-AuNP-MB biosensor probe was prepared by mixing 2 mL AuNP-SiO_2_-Qdots with 500 μl of 0.1 M EDC, 1.5 mL aqueous solution of the MB (0.4 μM in Tris-EDTA buffer) and 500 μl of 0.1 M NHS. The solution was stirred for ~1 h and stored at 4 °C prior to use.

### Fluorescence Detection Procedure

The detection of DNA was carried out under ambient condition. The SiO_2_-Qdots-AuNP-MB probe solution (5 μl) was mixed with 45 μl of Tris-EDTA buffer and 5 μl of the DNA target in a 96 well plate. Afterward, the solution was allowed to hybridize for ~3 min and the fluorescence was measured at a wavelength range of 480–800 nm with an excitation wavelength of 470 nm.

### Procedures for the ZP Analysis of the Thiol-capped AuNPs

Sample preparation is crucial in obtaining accurate data from the ZP analysis. Preserving the optical state of the thiol-capped AuNPs during the dilution process was our primary aim. Ideally, highly concentrated samples are not suitable for direct ZP measurements; hence, colloid solution of different thiol-capped Qdots was prepared from their stock solution in ultrapure deionized water and 800 μl of each sample solution was pipetted into a capillary cell (DTS 1060). All samples were prepared and analyzed on the same day. For each sample, the measurements were carried out in triplicate. Each sample analysis gives a mean ZP charge and a corresponding standard deviation.

### Detection Principle for DNA Detection

**Scheme 1:**
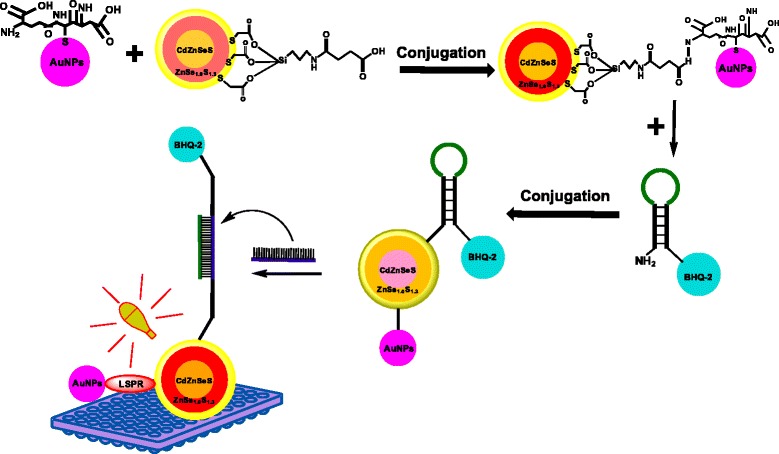
Schematic representation of the conjugation of GSH-AuNPs to SiO_2_-capped CdZnSeS/ZnSe_1.0_S_1.3_ Qdots, formation of the AuNP-SiO_2_ nanohybrid, further conjugation to MB and the LSPR-induced fluorescence detection of DNA

Scheme 1 shows the detection principle of the biosensor developed in this work. GSH-AuNPs were conjugated to SiO_2_-Qdots to form a plasmon-enhanced AuNP-SiO_2_-Qdots fluorescent nanohybrid. The quenching of the fluorescence of the AuNP-SiO2-Qdot after conjugation to the MB is due to the close distance with the BHQ-2 quencher. The hybridization of the target DNA with the loop sequence of the MB stretches the distance between AuNP-SiO_2_-Qdots and BHQ-2 and this effect tranduces LSPR signal from AuNPs to the Qdots, which afterward is reflected by the fluorescence turn-on intensity of the Qdots as a function of the DNA concentration.

## Results and Discussion

### TEM Analysis of the Thiol-capped AuNPs

**Fig. 1:**
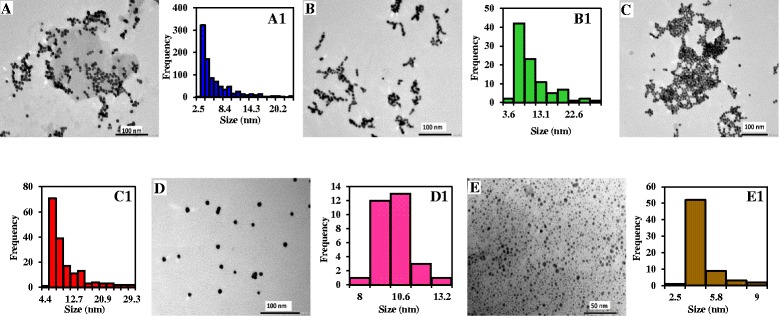
TEM images and corresponding particle size distribution histogram of **a**, **a1** TGA-AuNPs, **b, b1** MPA-AuNPs, **c, c1** l-cysteine-AuNPs, **d, d1** GSH-AuNPs, and **e, e1** cysteamine-AuNPs

TEM analysis was used to probe the morphology of the NPs and to determine the average particle size. Figure 1a–e shows the TEM images for the thiol-capped AuNPs. Generally, the particles are spherical in shape and relatively monodisperse. The synthetic method employed in this work for AuNPs is to produce spherical-shaped particles while the ligand exchange reaction is expected not to distort the morphology. Based on the TEM images, we affirm that the preparative method employed for the negatively charged thiol-capped AuNPs generated spherical-shaped NPs. The corresponding particle size distribution histogram is shown in Fig. 1a1–e1. The average size obtained is 5.9 nm for TGA-AuNPs, 9.0 nm for MPA-AuNPs, 9.2 nm for _L_-cysteine-AuNPs, 9.7 nm for GSH-AuNPs and 3.8 nm for cyteamine-AuNPs, respectively. Since the same citrate-capped AuNP solution was used for the preparation of the negatively charged thiol-capped AuNPs, the differences in size may be attributed to the variation in the number of thiol ligands anchored on the NP surface as well as in the bulkiness of the ligand. The bulkiness of the ligands and particle size of the negatively charged NPs follow the order: TGA < MPA < l-cysteine-AuNPs < GSH-AuNPs. It is therefore surprising that the order in bulkiness of the ligand corresponds to the order in the particle size variation. We anticipate this may directly explain the differences in particle size.

### UV/vis Absorption Analysis of the Thiol-capped AuNPs

**Fig. 2:**
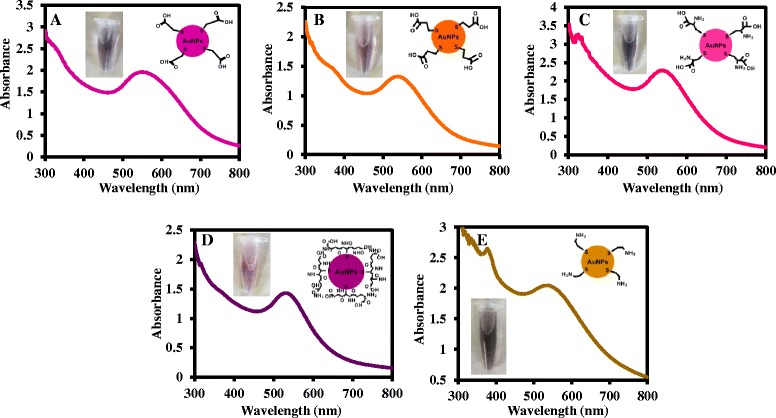
UV/vis absorption spectra of **a** TGA-AuNPs, **b** MPA-AuNPs, **c** l-cysteine-AuNPs, **d** GSH-AuNPs, and (**e**) cysteamine-AuNPs. *Inset*: the structure of the NPs and the color of the NP solution taken under ambient light

UV/vis absorption measurement is one of the most direct technique to confirm the synthesis of AuNPs. Figure 2a–e shows the typical surface plasmon resonance peaks in the absorption spectra for TGA-AuNPs, MPA-AuNPs, l-cysteine AuNPs, GSH-AuNPs, and cysteamine AuNPs. The plasmon peak of TGA-AuNPs absorbs at 548 nm, MPA-AuNPs absorbs at 536–538 nm, l-cysteine-AuNPs and cysteamine AuNPs absorbs at 534 nm, and GSH-AuNPs absorbs at 530 nm. Spherical AuNPs are generally known to have their plasmonic absorption within the visible region [24]. The difference in plasmonic absorption of the NPs is related to their surface capping ligand. The inset of each figure shows the difference in solution color of the NPs.

### DLS Analysis of the Thiol-capped AuNPs

**Fig. 3:**
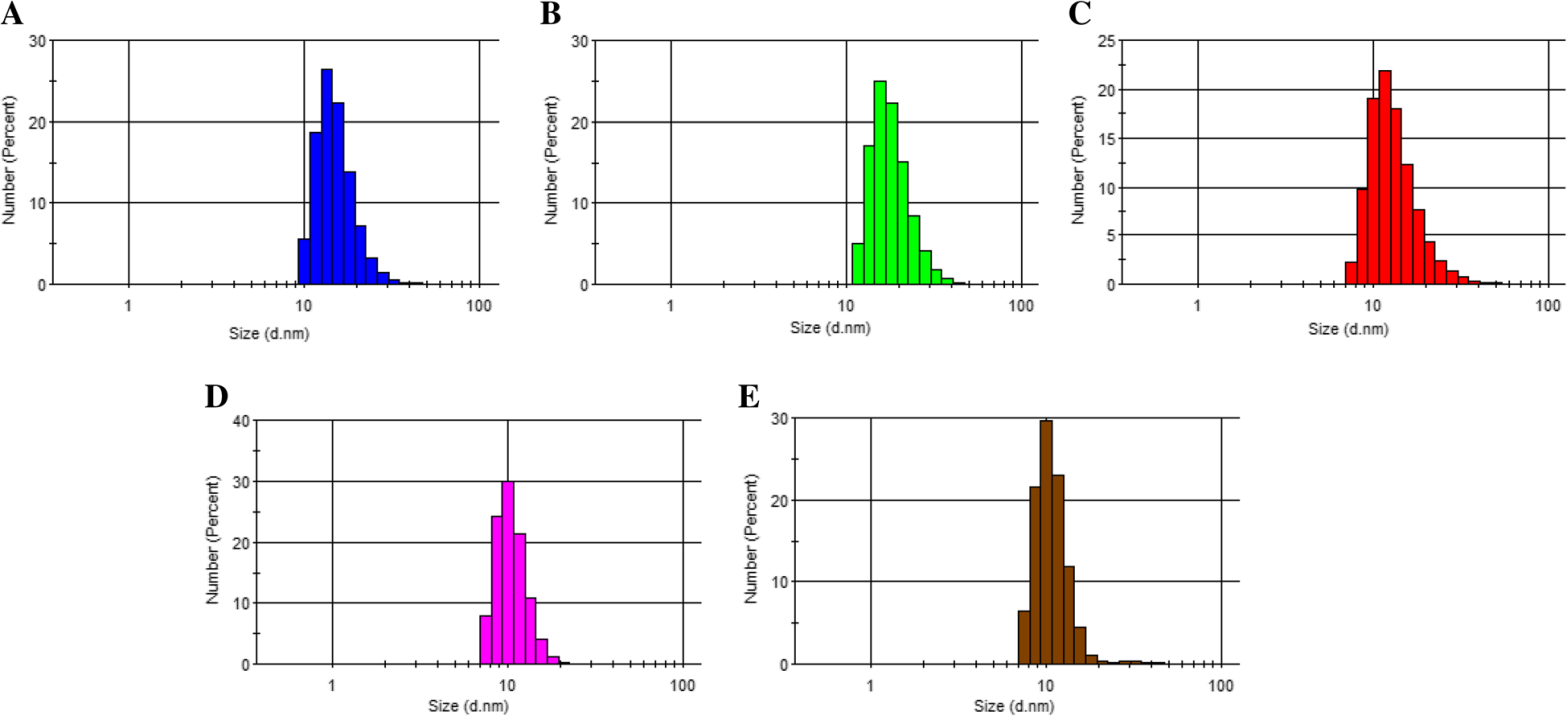
DLS histogram plots of **a** TGA-AuNPs, **b** MPA-AuNPs, **c** l-cysteine-AuNPs, **d** GSH-AuNPs, and **e** cysteamine-AuNPs

DLS was used as a technique to analyze the hydrodynamic particle size of the NPs. The basic operation of DLS relates to scattering of light by particles. The 6th power of the NP radii is directly proportional to the incident light scattered [25]. DLS then detects the intensity of the scattered light by the NP dispersion. The hydrodynamic size value can also be used as a measure to assess the aggregation state of the NP dispersion. Typically, NP dispersion with hydrodynamic size value <100 nm or close to the TEM value is considered to unaggregate, whereas NP dispersion with hydrodynamic size >100 is indicative of aggregation and reflects a high polydispersive index. Figure 3a–e shows the DLS curves of the thiol-capped NPs. The measured hydrodynamic size values are 15.51 ± 4.5 nm for TGA-AuNPs, 18.24 ± 4.9 nm for MPA-AuNPs, 13.60 ± 5.1 nm for l-cysteine-AuNPs, 10.64 ± 4.9 nm for GSH-AuNPs, and 10.83 ± 2.3 nm for cyteamine-AuNPs. The values obtained are relatively close to the TEM size values, hence confirming the monodispersity and unaggregated state of the NPs.

### ZP Theory

**Fig. 4:**
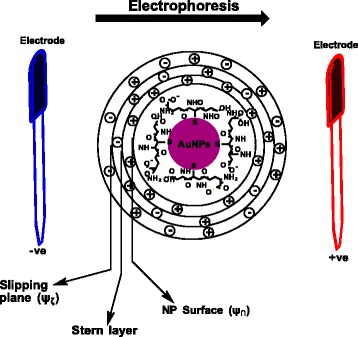
Schematic representation of the EDL process on GSH-AuNPs. The Stern layer is strongly anchored to the surface of the NP and consists of opposite charged ion. A diffuse layer then develops beyond the Stern layer and comprises of both positive and negative charges. In the process of electrophoresis, the NP with the adsorbed EDL migrates in the direction of the positive electrode while the slipping plane represents the interface between the dispersant and mobile NP. The ZP charge is deduced from the potential at the slipping plane. GSH-AuNPs are used as a representative NP for this description

Tangential flow of liquid along a charged colloidal surface can be induced by an applied mechanical force or by an external electric field (electro-osmosis, electrophoresis). Research has unravelled that a very thin layer of fluid anchors to the surface of the colloidal particle under the influence of the tangential motion [26]. The thin layer of fluid, called the hydrodynamic stagnant layer, stretches to a specified distance from the surface and reaches a hydrodynamic slipping plane that exists away from the particle surface. Electrokinetic potential, also known as ZP (ζ), is the potential at the shear/slipping plane of a particle surface moving under the influence of an applied electric field [27]. The potential difference between the layer of dispersant around the particle surface at the slipping plane and the electric double layer (EDL) of electrophoretic mobile particle is a reflection of the ZP. With respect to understanding the charged surface of NPs (Fig. 4), the spatial distribution of ions surrounding the charged surface is referred to as the EDL. The EDL layer is embedded with an excess of ions with opposite sign to the fixed charge (counterions) and contains a deficit of ions of the same sign to the fixed charge (co-ions). The uncharged region between the regime of hydrated counterion and the surface is known as the *Stern layer*, while the ions that are formed beyond this layer creates the *Gouy layer* or *diffuse layer* [28].

Direct measurement of ZP is impossible; hence, ZP data are conjectured under the influence of an applied electric field from electrophoretic mobility of the charged NP. The electrophoretic mobility (*μ*_e_), m^2^V^−1^s^−1^, is the magnitude of the electrophoretic velocity divided by the magnitude of the applied electric field strength. Positive electrophoretic mobility is characterized by the particle moving toward the negative electrode (lower potential) while negative mobility is characterized by the particle moving toward the positive electrode (higher potential) [28]. Electrophoretic mobility is calculated as Eq. 1 [27]:

1 $$ {\mu}_e=\frac{V}{E} $$

where *E* is the applied electric field strength (Volt/cm) and *V* is the particle velocity (μm/s). Henry’s formula can be used to deduce the ZP from *μ*_*e*_. This formula is applicable if the ZP is presumed to be less than 50 mV. Under this condition, concentration polarization and surface conductivity are regarded to be negligible. Henry postulated the expression for a nonconducting sphere as Eq. 2 [28]:

2 $$ {\mu}_e=\frac{2{\varepsilon}_{\mathrm{rs}}{\varepsilon}_0\zeta f1 ka}{3\eta } $$

where *η* is the dynamic viscosity of the experimental liquid, *f*_1_ *Ka* is the Henry’s function, *ε*_0_ is the electric permittivity of vacuum and *ε*_rs_ is the relative permittivity of the electrolyte solution. If the particle radius is larger than the thickness of the EDL as a result of particles reaching up to 1 μm within high salt concentrated aqueous dispersion solution (10^−2^ M), then the value of *f*_1_ *Ka* is taken as 1.5 while the Henry’s equation changes to the *Helmholtz-Smoluchowski* equation [27]:

3 $$ {\mu}_e=\frac{\varepsilon_r{\varepsilon}_0\zeta }{\eta } $$

whereas, if the particle radius is smaller than the EDL thickness as a result of low salt concentrated dispersion solution (10^−5^ M), *f*_1_ *Ka* is <1 and the Henry’s equation changes to the *Hückel-Onsager* equation as follows:

4 $$ {\mu}_e=\frac{2{\varepsilon}_{rs}{\varepsilon}_0\zeta }{3\eta } $$

Eq. 3 is applicable to pharmaceutical preparations for nano-drug delivery systems [29] while Eq. 4 is applicable in the ceramic industry [27].

Theoretically, the stability of NP dispersion is determined by the balance in repulsive and attractive forces [30]. If the attractive forces are lesser than the repulsive forces, then the NP dispersion remains stable. The thiol-capped AuNPs studied in this study are anchored with negatively charged carboxylate thiol ligands; hence, the electrostatic repulsion with respect to the bulkiness of the thiol ligand is expected to dominate in solution. Also, within the pharmaceutical domain, the ZP guidelines used in classifying colloidal NP dispersion in drug delivery systems are highly unstable (±0–10 mV), relatively stable (±10–20 mV), moderately stable (±20–30 mV), and highly stable (±30 mV), respectively [31]. We have used this guideline to assess the colloidal stability of the thiol-capped AuNPs and also to study trends in their ZP charge as a function of pH, ionic strength, and NP concentration.

### The Influence of pH on the ZP of the Thiol-capped AuNPs

**Fig. 5:**
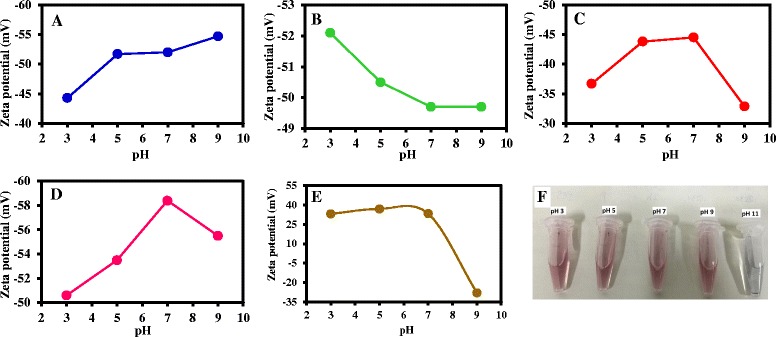
ZP plot as a function of pH for **a** TGA-AuNPs, **b** MPA-AuNPs, **c** l-cysteine-AuNPs, **d** GSH-AuNPs, and **e** cysteamine-AuNPs. pH = 3, 5, 7, and 9. **f** Color of GSH-AuNPs solution taken under ambient light at different pH

The most significant parameter in ZP analysis of aqueous colloidal dispersion in pharmaceutical formulations is perhaps the pH. Variation in ZP with respect to pH is reflected in the charge being more negative or positive in magnitude with basic or acidic pH [32]. Figure 5 shows the effects of pH on the ZP of TGA-AuNPs (Fig. 5a), MPA-AuNPs (Fig. 5b), l-cysteine-AuNPs (Fig. 5c), GSH-AuNPs (Fig. 5d), and cysteamine-AuNPs (Fig. 5e). The corresponding ZP charge is listed in Table 1 while Additional file 1: Figure S1 displays the ZP curves. The first direct observation is the lack of ZP data at pH 11 which is due to aggregation of the NP. Figure 5e shows the comparison in color of GSH-AuNP solution at the different pH, with the visible evidence of aggregation/flocculation and loss of chemical stability of the NP at pH 11. This result reveals that beyond pH 9, the NP becomes unstable in solution and hence reaches its isoelectric point. From the ZP data (Table 1), we can infer that each of the thiol-capped AuNPs was highly stable, but there was a variation in ZP charge within the regime of high colloidal stability (Fig. 5). Generally, with the exception of pH 11, the ZP charge was dependent on the pH. Since all the ZP charges are within the regime of high colloidal stability, we have based our assessment within this state. Within the pH range (pH 3–9) examined, the trend in the ZP charge was similar for l-cysteine-AuNPs and GSH-AuNPs but different for TGA-AuNPs and MPA-AuNPs. Results show a stronger ZP charge (more negative) at increasing pH for TGA-AuNPs, which reached a plateau at pH 9. Whereas for MPA-AuNPs, we observe a decrease in the strength of the ZP charge at increasing pH and reaching a plateau at pH 7. At pH 9, the ZP remained unchanged, thus indicating that the ZP charge is independent of pH. This indicates that higher colloidal stability is best at pH 3. For l-cysteine-AuNPs and GSH-AuNPs, the trend in ZP data was similar. The ZP charge was more strongly negative as the pH increased from 3 to 7 and decreased at pH 9, thus revealing that a plateau in higher colloidal stability was attained at pH 7. This implies that higher colloidal stability is best at pH 7 for l-cysteine-AuNPs and GSH-AuNPs.

**Table 1:** Effects of pH on the ZP of thiol-capped AuNPs

pH	TGA-AuNPs (mV)	MPA-AuNPs (mV)	l-cysteine-AuNPs (mV)	GSH-AuNPs (mV)	Cysteamine-AuNPs (mV)
3	−44.3 ± 11.4	−52.1 ± 8.9	−36.7 ± 10.5	−50.6 ± 14.6	+33.1 ± 8.1
5	−51.7 ± 13.4	−50.5 ± 8.6	−43.8 ± 10.2	−53.5 ± 10.3	+37.0 ± 8.0
7	−52.0 ± 11.5	−49.7 ± 16.4	−44.5 ± 8.9	−58.4 ± 15.8	+33.3 ± 6.8
9	−54.7 ± 13.9	−49.7 ± 14.0	−32.9 ± 12.4	−55.5 ± 11.0	−27.9 ± 7.9
11	–	–	–	–	

For the cationic cysteamine-AuNP, the ZP charge was positive between pH 3 and 7 but dramatically changed to negative at pH 9. This implies that between pH 3 and 7, the cysteamine ligand maintained its functionality as evidence from the strongly positive ZP charge which also indicated high colloidal stability. The transformation of the ZP charge from positive to negative at pH 9 indicates loss of chemical stability of the NP which may arise due to deprotonation of the thiol functional moiety or removal of the cysteamine functional group from the surface. We hereby recommend that the cationic AuNPs should be utilized between pH 3 and 7.

### The Influence of Ionic Strength on the ZP of the Thiol-capped AuNPs

**Fig. 6:**
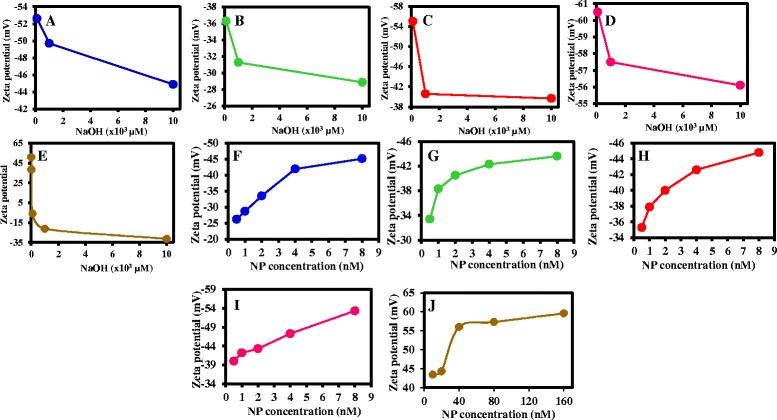
ZP plot as a function of ionic strength for **a** TGA-AuNPs, **b** MPA-AuNPs, **c** l-cysteine-AuNPs, **d** GSH-AuNPs and **e** cysteamine-AuNPs. [NaOH] = 0.1, 10, 10^2^, 10^3^, and 10^4^ μM (**a**–**e**). ZP plot as a function of NP concentration for **f** TGA-AuNPs, **g** MPA-AuNPs, **h** l-cysteine-AuNPs, **i** GSH-AuNPs, and **j** cysteamine-AuNPs.. NP concentration = 0.5, 1, 2, 4, and 8 nM (**f**–**i**) and 10, 20, 40, 80, and 160 nM (**j**)

Solution of NaOH in the concentration range of 10^2^–10^6^ μM was selected to study the effects of ionic strength on the ZP of the different thiol-capped AuNPs. Table 2 provides the ZP data, Fig. 6 shows the plots of the ZP charge as a function of the ionic strength, and Additional file 1: Figure S2 shows the overlay of the ZP curves. The first direct observation points to the lack of ZP data at 10^5^ and 10^6^ μM for the respective thiol-capped AuNPs. We can draw the conclusion that aggregation and loss of chemical stability occurred for the NPs at this ionic strength. Our results show that ZP data for thiol-capped AuNPs are viable between ≤10^4^ μM NaOH. Assessing the colloidal stability of the NPs based on their ZP charge obtained at 10^2^, 10^3^, and 10^4^ μM NaOH, TGA-AuNPs (Fig. 6a), l-cysteine-AuNPs (Fig. 6c), and GSH-AuNPs (Fig. 6d), exhibited high colloidal stability within these ranges of ionic strength. However, for MPA-AuNPs (Fig. 6b), the ZP charge (–28.9 ± 12.6 mV) at 10^3^ μM provides direct evidence of moderate colloidal stability while the ZP charge at 10 and 10^2^ μM was within the range of high colloidal stability. Comparing the extent of negativity of the ZP charge, we found GSH-AuNPs to be the most negative and MPA-AuNPs to be the less negative. From Fig. 6, we observed the same trend in ZP charge of the respective NPs. The plots show that less ionic strength concentration favors a more negative ZP and an increasing colloidal stability within the regime of high colloidal stability. Increasing ionic strength is known to compress the EDL which results in decrease of the ZP and vice versa [27] as we have observed in this work. Generally, our analysis has shown that ionic strength influenced the ZP charge of thiol-capped AuNPs.

**Table 2:** Effects of ionic strength on the ZP of thiol-capped AuNPs

NaOH (μM)	TGA-AuNPs (mV)	MPA-AuNPs (mV)	l-cysteine-AuNPs (mV)	GSH-AuNPs (mV)	Cysteamine-AuNPs (mV)
10^6^	–	–	–	–	
10^5^	–	–	–	–	
10^4^	−44.9 ± 11.7	−28.9 ± 12.6	−39.7 ± 14.0	−56.1 ± 16.4	−31.4 ± 12.1
10^3^	−49.7 ± 8.2	−31.3 ± 5.9	−40.6 ± 12.6	−57.5 ± 12.9	−21.2 ± 12.4
10^2^	−52.6 ± 13.2	−36.3 ± 15.0	−55.0 ± 16.9	−60.5 ± 13.0	−6.07 ± 4.6
10^1^	Not measured	Not measured	Not measured	Not measured	+38.5 ± 7.2
0.1	Not measured	Not measured	Not measured	Not measured	+50.9 ± 11.9

At 10^4^–10^2^ μM ionic strength, the cationic cysteamine-AuNP exhibited a decreasing negative ZP charge (Fig. 6e, Table 2), thus indicating the degree of deprotonation of the functional moiety on the surface of the NP. We found the NP to exhibit high colloidal stability at ionic concentration ≤10 μM. The strong ZP charge at 0.1 μM is indicative of a highly stable colloidal NP state. It is highly recommended that the cationic AuNPs should be utilized at a very low ionic strength, typically ≤10 μM.

### The Influence of NP Concentration on the ZP of the Thiol-capped AuNPs

The relationship between NP concentration and ZP charge is relatively complex and often determined by the effect of EDL and surface adsorption [27]. There are no general guidelines as to the effect of NP concentration on ZP. What can be inferred is that as the particle concentration increases, the ZP charge increases and vice versa. This is influenced by the thickness of the EDL and surface adsorption of the dilute colloidal dispersion solution [33]. Figure 6f–j shows the plot of the ZP charge against the NP concentration while Table 3 lists the ZP values (for the negative-charged thiol NPs) and Additional file 1: Figure S3 displays the ZP curves. The effects of NP concentration on the ZP charge were studied in the range of 0.5–8 nM for the negative-charged thiol NPs and from 10–160 nM for the cationic AuNP. As the concentration of the NP increased, we observed a direct increase in the ZP charge for all the thiol-capped AuNPs, TGA-AuNPs (Fig. 6f), MPA-AuNPs (Fig. 6g), l-cysteine-AuNPs (Fig. 6h) and GSH-AuNPs (Fig. 6i), and cysteamine-AuNPs (Fig. 6j). The ZP charge obtained for cysteamine-AuNP is +43.4 ± 11.6 mV (10 nM), +44.3 ± 30.2 mV (20 nM), +56.0 ± 18.s mV (40 nM), +57.3 ± 8.1 mV (80 nM), and +59.6 ± 8.7 mV (10 nM), respectively. With the exception of TGA-AuNPs which exhibited moderate colloidal stability at 0.5 and 1.0 nM, the rest of the ZP charge of the thiol-capped AuNPs were all in the range of high colloidal stability. The most important difference relates to the ZP charge for GSH-AuNPs being more negative than the rest of the NPs. This shows that the strength in ZP charge of GSH-AuNPs is higher than the rest of the negatively charged thiol NPs.

**Table 3:** Effects of NP concentration on the ZP of thiol-capped AuNPs

NP (nM)	TGA-AuNPs (mV)	MPA-AuNPs (mV)	l-cysteine-AuNPs (mV)	GSH-AuNPs (mV)
0.5	−26.2 ± 12.6	−33.4 ± 5.3	−35.3 ± 17.2	−40.0 ± 9.7
1.0	−28.7 ± 15.4	−38.3 ± 5.1	−37.9 ± 23.3	−42.2 ± 13.7
2.0	−33.5 ± 5.8	−40.5 ± 13.3	−40.0 ± 11.9	−43.3 ± 17.3
4.0	−41.9 ± 10.9	−42.3 ± 9.3	−42.6 ± 11.0	−47.3 ± 17.1
8.0	−45.1 ± 10.8	−43.6 ± 8.8	−44.8 ± 10.6	−53.3 ± 14.0

## Biosensor Application

### Characterization of SiO_2_-Qdots, SiO_2_-Qdots-AuNP, and SiO_2_-Qdots-AuNP-MB

**Fig. 7:**
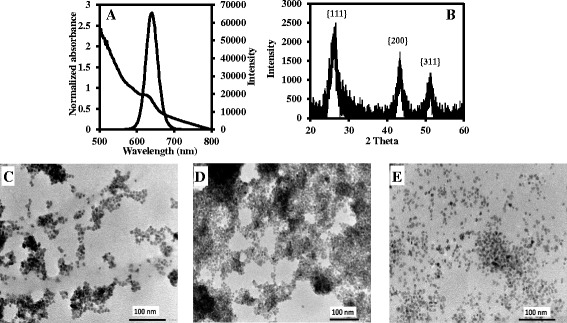
Characterization of SiO_2_-Qdots. UV/vis and fluorescence emission spectra (**a**), PXRD (**b**), and TEM image (**c**). TEM images of AuNP-SiO_2_-Qdots (**d**) and AuNP-SiO_2_-Qdots-MB (**e**)

Figure 7a shows the UV/vis absorption and fluorescence emission spectra of SiO_2_-Qdots. The excitonic absorption peak absorbs at 616 nm while the fluorescence maximum wavelength emits at 640 nm. A well-projected narrow full width at half maximum of 38 nm was exhibited by SiO_2_-Qdots, thus indicating homogenous nucleation and a monodisperse particle size distribution. The fluorescence quantum yield was calculated to be 78%. The corresponding PXRD, shown in Fig. 7b, projects a cubic crystalline zinc-blende crystal structure with three notable peaks at planes {111}, {220}, and {311}. The morphology of the SiO_2_-Qdots as shown in the TEM image (Fig. 7c) displays a quasi-spherical shape and slightly coarse particle size distribution. This particle morphology is due to the strong encapsulation of the silica layer. Discussion on the DLS and ZP plots of the SiO_2_-Qdots are presented Additional file 1: Figure S4.

The TEM images of AuNP-SiO_2_-Qdots nanohybrid and the AuNP-SiO_2_-Qdots-MB biosensor conjugate are shown in Fig. 7c, d. The strong coarseness of the particle morphology for AuNP-SiO_2_-Qdots nanohybrid is due to the strong binding of the AuNPs to the SiO_2_-Qdots whereas we observe a more monodisperse particle size distribution for the AuNP-SiO_2_-Qdots-MB conjugate due to the binding of the nanohybrid to the MB. Discussion on the ZP in probing the colloidal stability of AuNP-SiO_2_-Qdots and AuNP-SiO_2_-Qdots-MB is presented in Additional file 1: Figure S4.

### DNA Detection

**Fig. 8:**
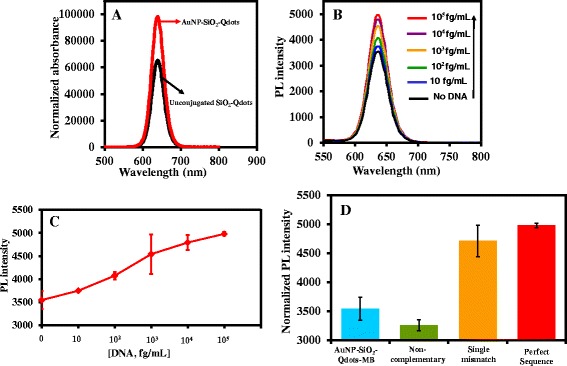
Plasmon-enhanced fluorescence effect of AnNPs on SiO_2_-Qdots upon binding (**a**), detection of perfect complementary nucleotide sequence (**b**), corresponding calibration curve (**c**), and specificity investigation of the biosensor (**d**)

Figure 8a shows the plasmon-enhanced fluorescence of AuNP-SiO_2_-Qdots nanohybrid. Due to the compact nature of the silica layer, the fluorescence of the Qdots is not quenched upon binding to AuNPs but rather enhanced. This implies that plasmonic effect of AuNPs induced fluorescence enhancement of the Qdots. The ultrasensitive fluorescence detection of a perfect complementary DNA sequence is shown in Fig. 8b while the corresponding calibration curve is shown in Fig. 8c. A wide concentration in the range of 10–10^5^ fg/mL was detected based on fluorescence enhancement changes of the AuNP-SiO_2_-Qdots-MB biosensor probe upon hybridization with the target DNA. Our LSPR-induced biosensor is not only sensitive to detect ultrasmall concentration of DNA but can also detect a wide range of concentration. The limit of detection (LOD) was calculated according to three times the standard deviation of blank measurement (*n* = 10) and dividing by the slope of the linear graph. The LOD of our biosensor for DNA was ~11 fg/mL which is equivalent to 1.4 fM. Table 4 shows the comparison of the LOD of our LSPR-induced biosensor system to published LOD for DNA using other types of Qdot-based biosensor probes. The comparison shows that our system is the most sensitive to date.

**Table 4:** Comparison of the LOD of the LSPR-induced Qdot-MB biosensor with other Qdot-MB-based probes

Probe name	LOD	Ref.
AuNP-SiO_2_-Qdots-MB	1.4 fM	This work
Thymine-DNA-Qdots-Hg^2+^-MB	25 nM	[34]
Qdots-microarray	0.1 pM	[35]
GSH-CdTe Qdot-MB	14 nM	[36]

### Distinguishing Single Nucleotide Mismatch and Noncomplementary Sequence

The efficacy of the biosensor to distinguish single nucleotide mismatch sequence and noncomplementary sequence from the perfect nucleotide sequence was investigated. As shown in Fig. 8d, the fluorescence enhancement effect at a fixed concentration of the targets 10^5^ (fg/mL) was slightly higher than for the perfect nucleotide sequence than for the single-base mismatch. However, for the noncomplementary sequence, the fluorescence was quenched. This demonstrates the specificity of our biosensor for the target nucleic acid.

## Conclusions

A novel LSPR-induced biosensor for DNA detection has been developed using an AuNP-SiO_2_-Qdots-MB biosensor. Prior to the detection of DNA, the colloidal stability as a function of the ZP charge for four negatively charged thiol-capped AuNPs and cationic AuNPs was investigated. Results showed that high colloidal stability was maintained for the negatively charged thiol-capped AuNPs from pH 3 to 9. However, for the cationic cyteamine-AuNPs, the colloidal stability was lost at pH > 7, thus indicating that high colloidal stability is maintained between pH 3 and 7. A strong dependence of the ZP charge on the ionic strength and NP concentration was observed for all the thiol-AuNPs. For the cationic cyteamine-AuNPs, it was unraveled that a very low ionic strength of ≤10 μM is needed to achieve high colloidal stability. A plasmon-enhanced AuNP-SiO_2_-Qdots nanohybrid was developed and further conjugated to a MB, thus forming an AuNP-SiO_2_-Qdots-MB biosensor. The AuNP-SiO_2_-Qdots-MB biosensor detected DNA down to 10 fg/mL based on LSPR-induced fluorescence signal while single-base nucleotide mismatch and noncomplementary sequence target were distinguished.

## Additional file

Additional file 1: Figures S1–S4. Supplementary information: **Figure S1.** Overlay of the ZP curves of (A) TGA-AuNPs, (B) MPA-AuNPs, (C) l-cysteine-AuNPs, (D) GSH-AuNPs and (E) cysteamine-AuNPs at pH 3, pH 5, pH 7 and pH 9; **Figure S2.** Overlay of the ZP curves of (A) TGA-AuNPs, (B) MPA-AuNPs, (C) l-cysteine-AuNPs (D) GSH-AuNPs and (E) cysteamine-AuNPs at different ionic strength; **Figure S3.** Overlay of the ZP curves of (A) TGA-AuNPs, (B) MPA-AuNPs, (C) l-cysteine-AuNPs and (D) GSH-AuNPs at different NP concentrations of 0.5, 1.0, 2.0, 4.0, and 8.0 nM; **Figure S4.** DLS and ZP plots for SiO_2_-capped CdZnSeS/ZnSe_1.0_S_1.3_ Qdots (A and B) and ZP plot for SiO_2_-Qdots-AuNP (C) and SiO_2_-Qdots-AuNP-MB (D). (DOCX 203 kb)
