# Supplementary material for: Gold Nanoparticle-Quantum Dot Fluorescent Nanohybrid: Application for Localized Surface Plasmon Resonance-induced Molecular Beacon Ultrasensitive DNA Detection
Source: Nanoscale Res Lett. 2016 Nov 25;11:523. doi: 10.1186/s11671-016-1748-3 (PMC5124020; doi:10.1186/s11671-016-1748-3)
Supplement: Additional file 1: Figures S1–S4. — Supplementary information: Figure S1. Overlay of the ZP curves of (A) TGA-AuNPs, (B) MPA-AuNPs, (C) l-cysteine-AuNPs, (D) GSH-AuNPs and (E) cysteamine-AuNPs at pH 3, pH 5, pH 7 and pH 9; Figure S2. Overlay of the ZP curves of (A) TGA-AuNPs, (B) MPA-AuNPs, (C) l-cysteine-AuNPs (D) GSH-AuNPs and (E) cysteamine-AuNPs at different ionic strength; Figure S3. Overlay of the ZP curves of (A) TGA-AuNPs, (B) MPA-AuNPs, (C) l-cysteine-AuNPs and (D) GSH-AuNPs at different NP concentrations of 0.5, 1.0, 2.0, 4.0, and 8.0 nM; Figure S4. DLS and ZP plots for SiO2-capped CdZnSeS/ZnSe1.0S1.3 Qdots (A and B) and ZP plot for SiO2-Qdots-AuNP (C) and SiO2-Qdots-AuNP-MB (D). (DOCX 203 kb) [file 11671_2016_1748_MOESM1_ESM.docx]

**Additional file 1**

**Gold Nanoparticle-Quantum Dot Fluorescent Nanohybrid: Application for Localized Surface Plasmon Resonance-induced Molecular Beacon Ultrasensitive DNA Detection**

Oluwasesan Adegoke,^1^ Enoch Y. Park^1,2,^^[[1]](#footnote-1)^

E-mail addresses:

adegoke.sesan@mailbox.co.za (OA),

park.enoch@shizuoka.ac.jp (EYP)

**
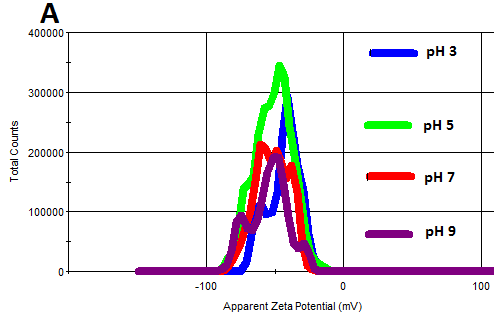
** **
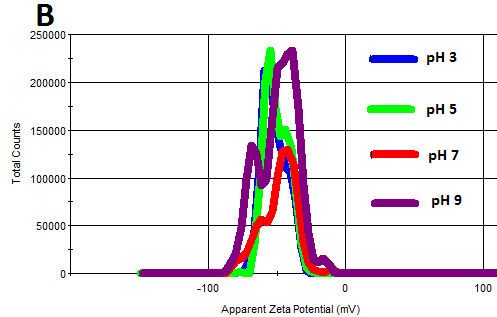
**

**
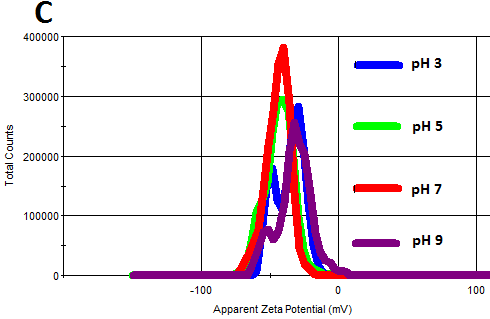
** **
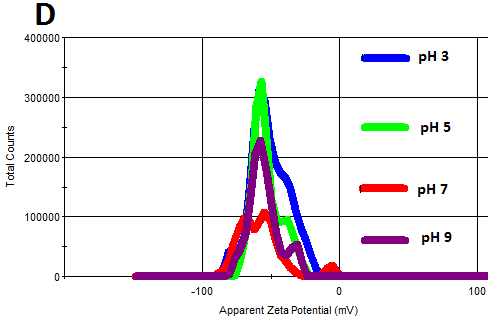
**


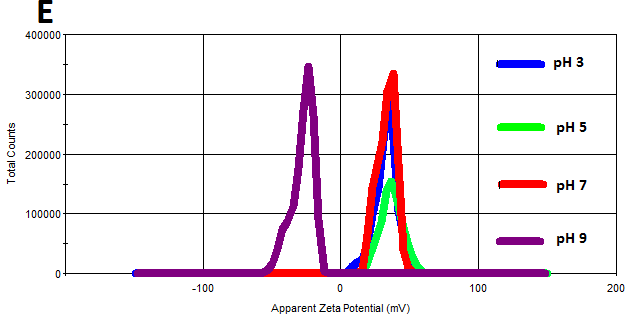


**Figure S1.** Overlay of the ZP curves of (A) TGA-AuNPs, (B) MPA-AuNPs, (C) _L_-cysteine-AuNPs, (D) GSH-AuNPs and (E) cysteamine-AuNPs at pH 3, pH 5, pH 7 and pH 9.


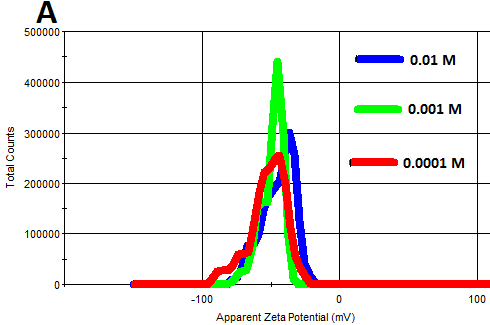

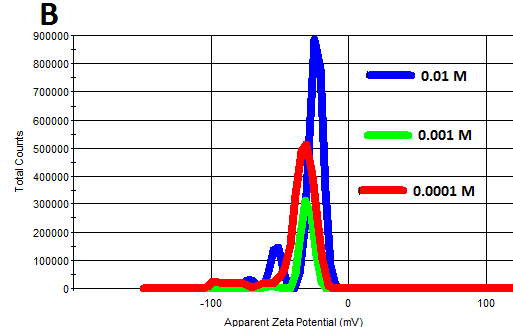


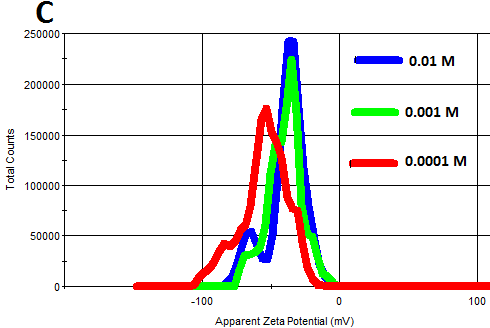

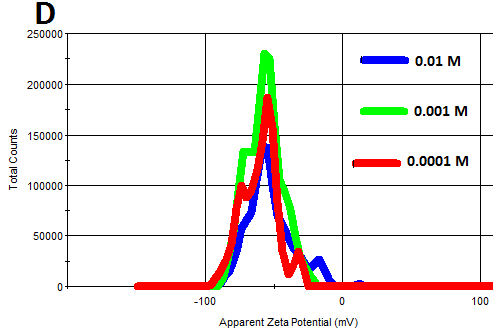


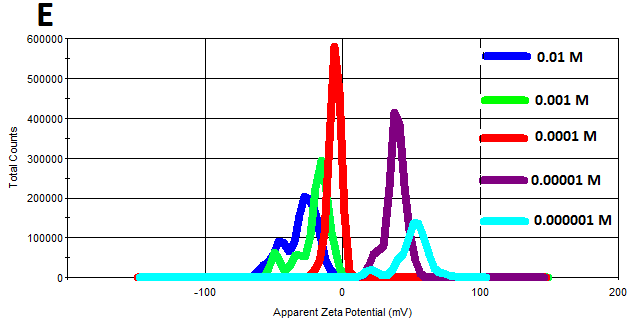


**Figure S2.** Overlay of the ZP curves of (A) TGA-AuNPs, (B) MPA-AuNPs, (C) _L_-cysteine-AuNPs and (D) GSH-AuNPs and (E) cysteamine-AuNPs at different ionic strength of 0.01 M, 0.001 M and 0.0001 M.


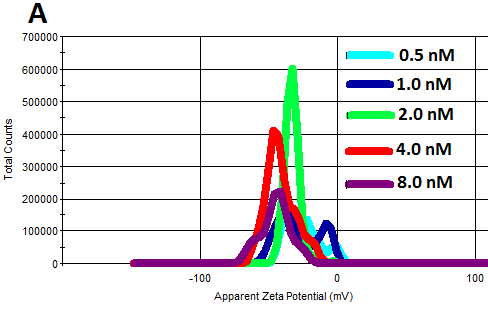

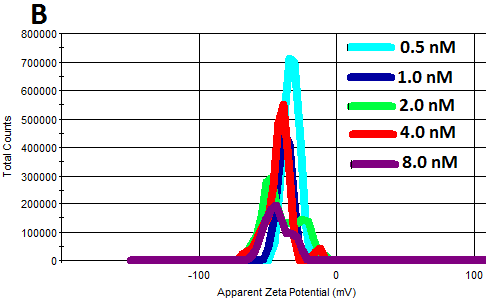


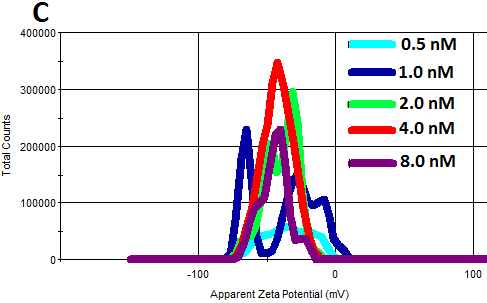

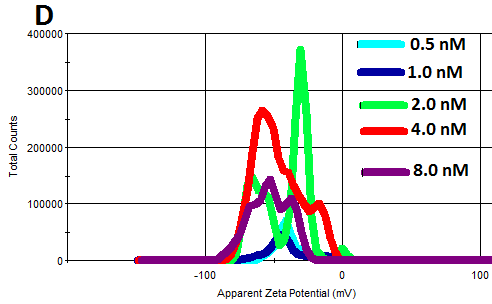


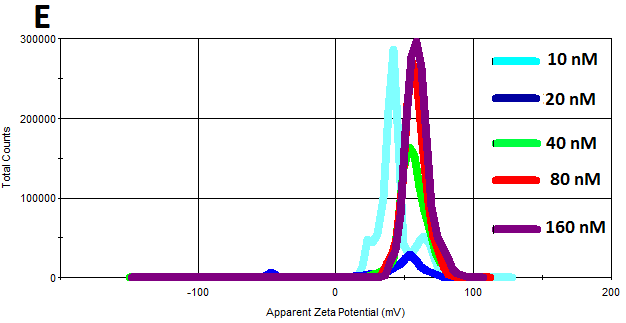


**Figure S3.** Overlay of the ZP curves of (A) TGA-AuNPs, (B) MPA-AuNPs, (C) _L_-cysteine-AuNPs (D) GSH-AuNPs and (E) cysteamine-AuNP at different NP concentration.

The DLS plot for the SiO_2_-Qdots as shown in Figure S4A exhibit a hydrodynamic particle size value of 52.2 nm which is well below 100 nm. This imply that the SiO_2_-Qdots is monodisperse and unagglomerated. The corresponding ZP charge as determined from the ZP plot (Figure S4B) was –38.0±5.3 mV, thus indicating a high colloidal stability of the SiO_2_-Qdots. ZP was also employed to probe the colloidal stability of the AuNP-SiO_2_-Qdots nanohybrid and the AuNP-SiO_2_-Qdots-MB biosensor. The ZP are shown in Figure S4C and D respectively. The values obtained are –34.5±3.9 mV for AuNP-SiO_2_-Qdots and –38.5±4.4 mV for AuNP-SiO_2_-Qdots-MB. The ZP charge values obtained indicate that high colloidal stability was retained by the nanohybrid and the biosensor conjugate.


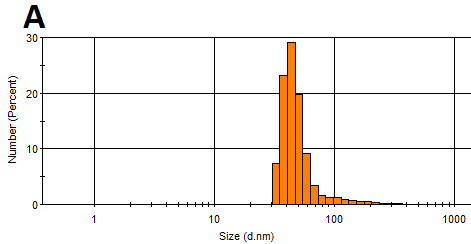

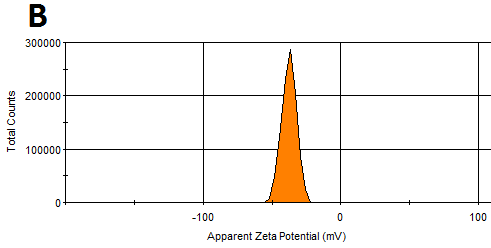


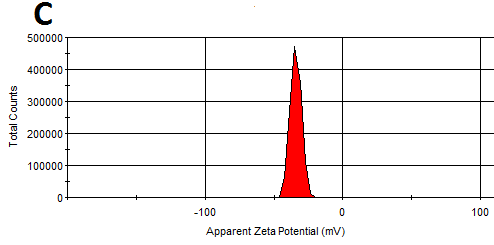

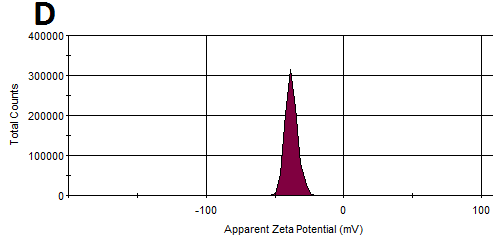


**Figure S4.** DLS and ZP plot for SiO_2_-capped CdZnSeS/ZnSe_1.0_S_1.3_ Qdots (A and B) and ZP plot for SiO_2_-Qdots-AuNP (C) and SiO_2_-Qdots-AuNP-MB (D).

1. Correspondence: park.enoch@shizuoka.ac.jp (EYP).

   ^1^ Laboratory of Biotechnology, Research Institute of Green Science and Technology, Shizuoka University, 836 Ohya, Suruga-ku, Shizuoka 422-8529, Japan

   ^2^ Laboratory of Biotechnology, Department of Bioscience, Graduate School of Science and Technology, Shizuoka University, 836 Ohya, Suruga-ku, Shizuoka 422-8529, Japan [↑](#footnote-ref-1)
